# QTc Prolongation as a Diagnostic Clue in Acute Pulmonary Embolism

**DOI:** 10.3390/jcm14145005

**Published:** 2025-07-15

**Authors:** Saleh Sharif, Eran Kalmanovich, Gil Marcus, Faina Tsiporin, Sa’ar Minha, Michael Barkagan, Itamar Love, Shmuel Fuchs, Guy Zahavi, Anat Milman

**Affiliations:** 1 Division of Cardiovascular Medicine, Shamir Medical Center, Beer Yaakov 70300, Israel; salehsharif6@gmail.com (S.S.); kalmanovicheran@gmail.com (E.K.); gilmarcus.gm@gmail.com (G.M.); fainatsif@gmail.com (F.T.); minha.saar@gmail.com (S.M.); barkagan@gmail.com (M.B.); loveitamar@gmail.com (I.L.); shmulik.fuchs@gmail.com (S.F.); 2 Faculty of Medical and Health Sciences, Tel Aviv University, Tel Aviv 6997801, Israel; guy@guyzahavi.com; 3 Department of Anesthesia and Intensive Care, Raphael Hospitals, Atidim Park, Building No. 3, Tel Aviv 6158101, Israel

**Keywords:** pulmonary embolism, QT prolongation, treatment, right ventricular dysfunction, acute PE, electrocardiography

## Abstract

**Background:** Pulmonary embolism (PE) increases right ventricular (RV) afterload, potentially leading to myocardial stress and electrocardiographic abnormalities. Although QTc prolongation has been suggested as a marker of RV dysfunction, its prevalence, clinical significance, and prognostic value in acute PE remain poorly defined. **Objective:** The objective of this study is to evaluate the prevalence and clinical implications of QTc prolongation in patients with intermediate–high and high-risk acute PE. **Methods**: We retrospectively analyzed 95 consecutive patients admitted with intermediate–high or high-risk PE between September 2021 and December 2023. QTc prolongation was defined as ≥470 ms in males and ≥480 ms in females. Clinical, imaging, and laboratory data were compared between patients with normal and prolonged QTc intervals. QTc was assessed at admission, after treatment, and prior to discharge. **Results:** QTc prolongation was observed in 28.4% of patients at presentation. This group had significantly higher lactate levels (2.3 vs. 1.8 mmol/L, *p* = 0.03) and a non-significant trend toward elevated troponin and lower oxygen saturation. No differences were observed in echocardiographic or CT-based RV dysfunction parameters. QTc values normalized by discharge irrespective of treatment modality. There was no association between QTc prolongation and in-hospital or long-term mortality. A trend toward more aspiration thrombectomy was noted in the prolonged QTc group (29.6% vs. 11.8%, *p* = 0.06). **Conclusions:** QTc prolongation is common in acute intermediate–high and high-risk PE and may reflect transient myocardial stress. While not predictive of clinical outcomes, it should be considered in the differential diagnosis of QTc prolongation in patients presenting with dyspnea and chest pain.

## 1. Introduction

Pulmonary embolism (PE) is the third most frequent acute cardiovascular syndrome worldwide, following myocardial infarction and stroke. Its annual incidence ranges from 39 to 115 cases per 100,000 population. PE is associated with high early mortality, accounting for up to 300,000 deaths annually in the United States. Approximately 34% of patients die suddenly or within a few hours of the acute event, often before treatment can be initiated [1]. Acute PE triggers a sudden increase in pulmonary vascular resistance (PVR), leading to right ventricular (RV) dilation, increased wall tension, and myocardial stretching. This sequence of pathophysiological events prolongs RV contraction, causing hemodynamic stress and potential cardiomyocyte damage if the oxygen supply fails to meet the elevated demand [2,3]. Although RV infarction is uncommon following PE, the disruption in the balance between oxygen delivery and myocardial demand can lead to ischemic injury [4,5]. Increased RV afterload, with its concomitant rise in wall stress, significantly elevates myocardial oxygen consumption while impeding perfusion, thereby exacerbating the mismatch between oxygen supply and demand. This imbalance, compounded by systemic hypoxia frequently observed in PE, can lead to myocardial injury, ischemia, and further compromise of RV contractility [6,7].

Electrocardiographic (ECG) manifestations of acute pulmonary embolism (PE) mainly reflect right-ventricular (RV) strain. In severe cases, they include T-wave inversion in V1–V4, a QR complex in V1, the S1Q3T3 pattern, and incomplete or complete right bundle branch block. In milder presentations, sinus tachycardia is often the sole abnormality, observed in approximately 40% of cases. Atrial arrhythmias, particularly atrial fibrillation, are also frequent [8,9,10]. These patterns arise from delayed right-sided conduction and repolarization disturbances produced by abrupt RV pressure overload, transient ischemia, and activation of stretch-sensitive ion channels. Notably, 10–25% of patients with acute PE have a normal ECG [10]; the absence of abnormalities, therefore, does not exclude the diagnosis.

QT interval prolongation in the setting of PE has been scarcely reported, with findings primarily published in a limited number of case studies. However, a recent study highlighted its prognostic importance in acute PE, showing that QT prolongation is associated with more extensive RV dilation, increased systolic dysfunction, and, consequently, longer hospital stays [11]. This subgroup of patients with prolonged QT intervals also demonstrated higher-risk PE, often requiring thrombolytic therapy. Despite these findings, the short- and long-term prognostic implications of QT prolongation in acute PE remain unexplored.

Risk stratification during the initial evaluation of PE patients is essential for guiding therapeutic decisions and optimizing outcomes [1,12]. However, primary risk assessment models typically do not incorporate ECG parameters, which could provide valuable prognostic insights. The aim of this study was to investigate the significance of QTc interval prolongation in acute intermediate–high and high-risk PE. Our specific objectives were to (i) determine the prevalence of QTc prolongation in this cohort; (ii) compare the clinical presentation, laboratory findings (including cardiac biomarkers), and imaging parameters of patients with prolonged versus normal QTc; and (iii) evaluate any association between QTc prolongation and management strategies or outcomes.

## 2. Materials and Methods

### 2.1. Study Design

This retrospective study included consecutive patients diagnosed with intermediate–high or high-risk PE who were admitted to the cardiology wards at Yitzhak Shamir Medical Center, a tertiary care university hospital in Israel’s Central District, from September 2021 to December 2023. Clinical, imaging, and laboratory data were collected at admission, throughout hospitalization, and at discharge. The study protocol was approved by the Institutional Review Board of Yitzhak Shamir Medical Center (0209-23-ASF, 14/09/2023).

### 2.2. Patient Identification and Main Measurements

Patients were identified through a systematic review of institutional service registers and medical records, ensuring data accuracy and completeness. Eligible patients were those with a confirmed diagnosis of acute PE classified as intermediate–high or high risk according to the 2019 European Society of Cardiology (ESC) guidelines [1], who underwent transthoracic echocardiography (TTE) within 24 h of admission and had at least three ECGs (at admission, after intervention, and one before discharge). Exclusion criteria included patients with acute PE not classified as intermediate–high or high risk, patients under 18 years of age, history of congenital long QT syndrome, incomplete ECG data at required time points, and patients admitted primarily for reasons other than PE. Notably, we did not exclude patients on medications known to prolong the QT interval; rather, such medications were documented for each patient (Table 1) and considered in our analysis. Additionally, we recorded admission potassium, magnesium, and calcium levels for each patient to account for electrolyte-related effects on QTc (Table 2).

### 2.3. PE Diagnosis

At presentation, patients with suspected PE underwent initial diagnostic evaluation via CT angiography (CTA), with concurrent assessments of laboratory markers for myocardial injury and clinical signs of shock. In our cohort, PE diagnosis was confirmed by CTA in all cases (demonstrating an intraluminal filling defect in the pulmonary arterial tree); no patient required alternate imaging such as a ventilation–perfusion scan for confirmation. All patients underwent an ECG and comprehensive echocardiographic examination within 24 h of PE diagnosis. Echocardiographic parameters assessed included RV size, tricuspid annular plane systolic excursion (TAPSE), TR severity, IVC diameter and collapse, and estimated pulmonary artery pressure. Tricuspid annular peak systolic velocity (TAPSV) and the presence of interventricular septal shift were also recorded as indicators of acute RV strain. A multidisciplinary Pulmonary Embolism Response Team (PERT), comprising critical cardiac care and interventional cardiology specialists, performed risk stratification using the simplified Pulmonary Embolism Severity Index (sPESI) [13] and the Society of Cardiovascular Angiography and Intervention (SCAI) score [14], determining an appropriate management strategy for each patient.

### 2.4. Treatment Allocation

Treatment options included anticoagulation therapy in the noninvasive group and percutaneous interventions in the invasive group. Percutaneous treatments involved catheter-directed thrombolysis using the EKOS™ endovascular system (Boston Scientific, Marlborough, MA, USA) and aspiration thrombectomy using devices such as the FlowTriever (Inari Medical, Irvine, California, USA) or the Lightning Indigo system (Penumbra Inc., Alameda, California, USA). Additionally, some patients initially classified as high-risk PE received systemic TPA according to guideline-based criteria; some of these patients were subsequently managed conservatively, while others proceeded to invasive treatment.

### 2.5. Electrocardiographic Analysis

A standard 12-lead ECG was usually recorded at three time points: upon presentation (typically in the emergency department before a confirmed diagnosis of PE), during hospitalization following treatment initiation, and prior to discharge. ECGs were recorded at a paper speed of 25 mm/s and an amplitude of 10 mm/mV. QTc measurements were performed independently by two experienced electrophysiologists (A.M. and M.B.), with subsequent consensus adjudication. The QT interval was measured manually from the onset of the QRS complex to the end of the T wave using the tangent method and corrected to the preceding R-R interval (QTc). Lead II served as the primary lead, with lead V5 used if lead II was unsuitable; if neither was suitable, an alternative lead was selected. We defined significant QTc prolongation as exceeding the 99th percentile threshold (QTc ≥ 470 msec for males and ≥480 msec for females).

### 2.6. Clinical Outcomes and Follow-Up

Patients were followed up for at least four months after discharge. Mortality data were obtained from the Ministry of Interior by the end of April 2025.

### 2.7. Statistical Analysis

Categorical variables are presented as counts and percentages. The distribution of continuous variables was assessed using the Shapiro–Wilk test and found to be non-normal; therefore, continuous variables are reported as medians with interquartile ranges. Group comparisons were performed using the Fisher exact test for categorical variables and the Kruskal–Wallis test for continuous variables.

Missing data on medical diagnoses were treated as indicating absence (‘No’). A two-tailed *p*-value < 0.05 was considered statistically significant. Multivariable analyses were not conducted due to the relatively small sample size. All analyses were performed using R version 4.4.0 (R Foundation for Statistical Computing, Vienna, Austria).

## 3. Results

### 3.1. Study Population

A total of 95 consecutive patients met the inclusion criteria over the two-year study period. The median age was 71 [55.5–77] years, with a slightly higher female distribution (Table 1).

The median Charlson Comorbidity Index was 4 [2–6]; comorbidities are listed in Table 1. Most patients presented to the emergency department with a SCAI score of A, with a high sPESI score, normal blood pressure, tachycardia, and slight hypoxia (Table 2). Laboratory results indicated elevated troponin T levels (median 101 [42.2–252] ng/L), elevated NT-proBNP levels (median 2157 [575–5328] pg/mL), and median lactate levels of 2.1 [1.3–3.4] mmol/L.

Imaging findings from CT and echocardiography are summarized in Table 3, with most patients presenting with a main pulmonary artery thrombus (76.8%).

QTc interval measurements at presentation showed mild prolongation, with a median QTc of 463 [442–492] milliseconds (msec) (Table 4).

### 3.2. QT Interval Analysis

Using the 99th percentile to define significant QTc prolongation (QTc ≥ 470 ms for males and ≥480 ms for females) in the whole study group, 28.4% exhibited a prolonged QTc. No differences were observed between QTc groups at admission, except for more dementia in the prolonged QTc group (*p* = 0.05, Table 1) and a higher lactate level at admission (2.3 [1.7–4.9] mmol/L vs. 1.8 [1–3.1] mmol/L in the normal QTc group, *p* = 0.03, Table 2). The prolonged QTc group tended toward greater myocardial injury and hypoxemia, as reflected by a higher median troponin T level (109 [68.2–275.8] ng/L vs. 94 [34.5–218] ng/L in the normal QTc group) and a lower median oxygen saturation on room air (90.5% vs. 95%), although these differences were not statistically significant (*p* = 0.27 and *p* = 0.11, respectively; Table 2). Imaging studies with CTA and echocardiography demonstrated no difference between the QT groups.

### 3.3. Treatment

As determined by the PERT, patients with intermediate–high or high-risk acute PE were managed with either noninvasive treatment (46 patients) or invasive procedures (49 patients). Table 5 describes the different treatment allocations. No significant difference was observed between the treatment allocation for the normal and prolonged QTc groups; however, there was a trend towards more aspiration in the prolonged QTc group (*p* = 0.06).

**Figure 1:**
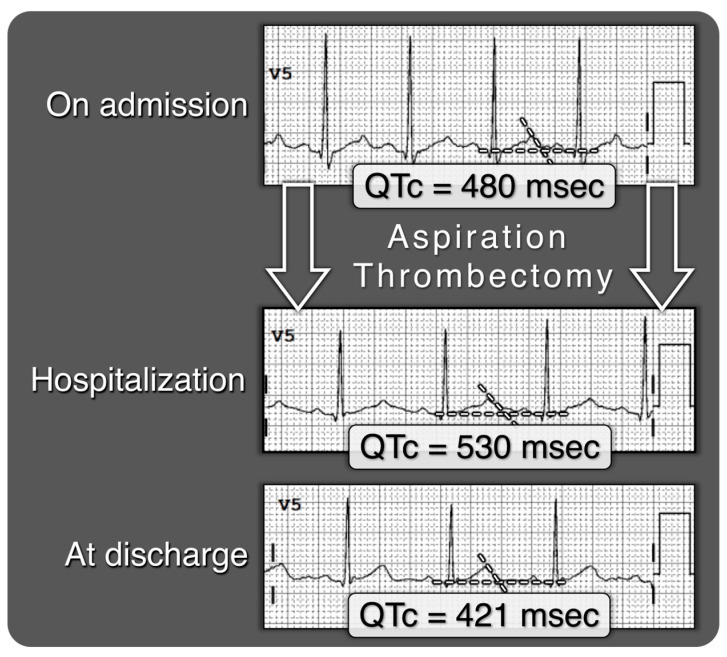
Dynamic QTc interval changes following aspiration thrombectomy in a patient with acute pulmonary embolism.

Interestingly, patients who were admitted with a significantly prolonged QTc (median 500 [491.1–520.2] vs. 450 [431.9–467.9], *p* < 0.001), normalized their QTc post-treatment and were not different at discharge from the non-prolonged group (Table 4, Figure 1), irrespective of invasive or noninvasive treatment allocation.

Serial 12-lead ECGs (lead V5 shown) from a patient with acute pulmonary embolism demonstrate progressive changes in the corrected QT interval (QTc) during hospitalization. On admission, the QTc was 480 ms. Following aspiration thrombectomy, the QTc was further prolonged to 530 ms, then normalized to 421 ms by discharge. These temporal changes highlight the transient nature of repolarization abnormalities in the acute phase of pulmonary embolism.

### 3.4. Outcomes

Hospitalization days were similar for both the normal QTc and prolonged QTc groups (6 [4–10.2] vs. 8 [4.5–11.5] days, respectively, *p* = 0.48). During hospitalization, five patients died, four with a normal QTc and one from the prolonged QTc group, all because of a massive PE complicated by cardiogenic shock. Mortality rates did not significantly differ between the treatment groups during this period, and no additional deaths occurred by 3-month follow-up.

Over six months of follow-up, two additional deaths occurred, both in the prolonged QTc group. During the 12-month follow-up, four more patients died, all from the normal QTc group. The causes of death during the 6- and 12-month follow-up periods were unknown. At all assessed time points, no significant differences in mortality were observed between the two groups (Table 6).

## 4. Discussion

In this retrospective study of patients with intermediate–high and high-risk acute pulmonary embolism (PE), we investigated the prevalence, clinical correlates, and prognostic value of QTc prolongation. We found that QTc prolongation was present in 28.4% of patients at presentation, a frequency higher than previously reported, and that it was associated with transient physiological derangements, including higher lactate levels and trends toward elevated troponin and lower oxygen saturation. Importantly, QTc values normalized by discharge in most patients, regardless of treatment strategy, suggesting that QTc prolongation in PE reflects an acute, reversible electrophysiologic response to myocardial stress rather than a persistent repolarization abnormality.

While earlier studies suggested that QTc prolongation may correlate with disease severity, RV dysfunction, or worse outcomes in PE [11], we did not find significant differences between QTc groups in echocardiographic or CT-based RV strain parameters, including RV/LV ratio, pulmonary artery pressure, or degree of tricuspid regurgitation. Moreover, no differences were observed in hospitalization duration or mortality at any time point during the 12-month follow-up. Notably, the cohort studied by Buppajarntham et al. [11] in 2014 encompassed pulmonary embolism patients across a broad spectrum of risk categories, in contrast to our study, which was restricted to individuals with intermediate–high and high-risk PE. This fundamental difference in study populations may account for the observed prognostic significance of QTc prolongation in their analysis, which was not replicated in our findings.

Similarly, a widened frontal QRS-T angle on the ECG has recently been associated with more severe RV dysfunction and adverse outcomes in acute PE [15], highlighting that other ECG markers can carry prognostic significance even if QTc itself does not. These findings argue against the independent prognostic utility of QTc prolongation in this patient population. However, our sample size was modest, and the study may not have been powered to detect very subtle outcome differences; thus, a small prognostic influence of QTc prolongation cannot be entirely ruled out without larger studies. A notable finding was the trend toward increased use of aspiration thrombectomy in patients with prolonged QTc (29.6% vs. 11.8%, *p* = 0.06), which may reflect clinicians’ perception of higher disease burden.

Our data also carries practical implications for emergency department evaluation. In patients presenting with chest pain and dyspnea, QTc prolongation is frequently attributed to more common causes such as electrolyte disturbances, ischemia, or medication effects. QTc prolongation is a well-recognized phenomenon in acute myocardial infarction (AMI), and has been reported in a notable proportion of patients with ST-elevation MI, where it is associated with worse clinical outcomes [16,17], and it also accompanies other acute cardiac conditions like myocarditis, in which approximately one-quarter of patients exhibit a prolonged QTc (often predictive of a worse outcome) [18]. However, in acute PE, this finding has been less well characterized; earlier reports estimated an incidence of only 10–20% [11]. Our finding of a 28.4% incidence in a higher-risk cohort underscores that PE should be considered in the differential diagnosis of QT prolongation in the acute setting, particularly when accompanied by hypoxia, elevated lactate, or suggestive clinical features. Although nonspecific, QT prolongation in this context may provide an early physiologic clue prompting further evaluation for PE. For instance, normalization of vital signs does not eliminate the possibility of acute PE [19], underscoring the importance of maintaining suspicion for PE even when initial instability resolves.

Finally, we observed that patients with prolonged QTc at baseline (median 500 ms) exhibited significant QTc shortening following treatment, such that QTc values at discharge were not statistically different from those with normal baseline QTc. This dynamic behavior reinforces the idea that QTc prolongation in PE is a marker of acute and reversible myocardial stress, potentially linked to transient ischemia or RV overload. However, its lack of correlation with clinical outcomes suggests that while QTc may have diagnostic value, it should not currently be used as a prognostic tool. Notably, emerging computational tools may further enhance risk assessment: an artificial intelligence-based ECG analysis recently showed a high negative predictive value and an area under the curve of ~0.84 for identifying high-risk acute PE [20], indicating that advanced ECG analysis could aid in early detection and stratification of severe PE cases.

From a management standpoint, the presence of QTc prolongation in a patient with acute PE should prompt clinicians to be cautious about adding other QT-prolonging medications (to minimize proarrhythmic risk), but it does not necessitate any alteration in PE-specific therapy. Notably, none of our patients developed torsades de pointes or other malignant arrhythmias; thus, the QTc prolongation observed appears to reflect acute strain rather than an impending arrhythmic syndrome. In practice, QTc prolongation in the context of PE is best viewed as an important diagnostic clue rather than as a contraindication to standard treatments or a trigger for aggressive antiarrhythmic measures.

## 5. Conclusions

QTc prolongation is observed in nearly one-third of patients with intermediate–high and high-risk acute PE and appears to reflect transient physiologic stress rather than irreversible myocardial injury. Although our study did not demonstrate an independent association with imaging-defined RV dysfunction or adverse clinical outcomes—possibly due to limited sample size—QTc prolongation may still serve as a supportive diagnostic marker in the emergency setting, particularly when PE is part of the differential diagnosis in patients presenting with dyspnea, chest pain, and a prolonged QT interval. Future studies are needed to assess whether incorporating QTc dynamics into clinical evaluation can improve early risk stratification or diagnostic precision in acute PE. In current practice, unexplained QTc prolongation should raise clinical suspicion for PE when consistent with the overall presentation.

## 6. Future Directions

Large-scale prospective studies should further evaluate the clinical significance of QTc prolongation in acute PE. In particular, future research should confirm whether QTc changes have prognostic value in larger cohorts and whether dynamic QTc trends can aid in risk stratification. It would also be valuable to investigate the mechanistic basis of QTc prolongation in PE—for example, correlating repolarization changes with biomarkers of myocardial injury or detailed imaging of RV strain. Advanced analytical tools, such as machine learning applied to ECG data, may improve the detection of acute PE or identification of high-risk patients. Ultimately, integrating QTc interval monitoring into PE management algorithms (in combination with established clinical scores) is an intriguing prospect that warrants exploration in future trials.

## 7. Limitations

This study is limited by its retrospective design, single-center setting, and modest sample size, which may reduce power to detect subtle associations or differences in long-term outcomes. Manual QTc measurement, though conducted by experienced electrophysiologists, introduces potential inter-observer variability. Potential contributors to QTc prolongations, such as electrolyte levels, concomitant medications, and autonomic tone, were not systematically collected and may have confounded results. Finally, QTc dynamics were not assessed beyond hospital discharge, limiting our understanding of longer-term electrophysiological trends. Due to the limited sample size, multivariate regression analysis was not performed; as a result, potential confounding variables could not be adjusted for in the outcome model.

## Figures and Tables

**Table 1:** Patients’ characteristics at baseline.

	Total	Normal QTc	Prolonged QTc	*p*-Value
**N (%)**	95	68 (71.6)	27 (28.4)	
**Age (Median [IQR])**	71 [55.5–77]	71 [53.2–77]	71 [62–78]	0.67
**Gender (M/F)**	43 (45.3)/52 (54.7)	30 (44.1)/38 (55.9)	13 (48.1)/14 (51.9)	0.82
**MI**	11 (11.6)	9 (13.2)	2 (7.4)	0.72
**CHF**	7 (7.4)	5 (7.4)	2 (7.4)	1
**PVD**	5 (5.3)	4 (5.9)	1 (3.7)	1
**CVA**	7 (7.4)	5 (7.4)	2 (7.4)	1
**Dementia**	6 (6.3)	2 (2.9)	4 (14.8)	0.05
**COPD**	16 (16.8)	12 (17.6)	4 (14.8)	1
**Diabetes Mellitus**	27 (28.4)	20 (29.4)	7 (25.9)	0.81
**CKD**	7 (7.4)	7 (10.3)	0 (0)	0.19
**Hypertension**	56 (58.9)	41 (60.3)	15 (55.6)	0.82
**Charlson Index (Median [IQR])**	4 [2–6]	4 [1.8–5]	4 [2–6]	0.61
**Drugs that prolong QT n(%)**	29 (30.5)	19 (27.9)	10 (37)	0.461

n(%) unless otherwise indicated; M/F: male/female; MI: myocardial infarction; CHF: congestive heart failure; PVD: peripheral vascular disease; CVA: cerebrovascular accident; COPD: chronic obstructive pulmonary disease; CKD: chronic kidney disease.

**Table 2:** Clinical and laboratory presentation.

	Class	Total	Normal QTc	Prolonged QTc	*p*-Value
**N (%)**		95	68 (71.6)	27 (28.4)	
**SCAI Score**	A	53 (55.8)	39 (57.4)	14 (51.9)	0.73
	B	31 (32.6)	22 (32.4)	9 (33.3)	
	C	5 (5.3)	4 (5.9)	1 (3.7)	
	D	2 (2.1)	1 (1.5)	1 (3.7)	
	E	4 (4.2)	2 (2.9)	2 (7.4)	
**sPESI high**		65 (68.4)	47 (69.1)	18 (66.7)	0.81
**Systolic BP**		126 [111–146]	126 [110.5–143.5]	126.5 [113.2–147.5]	0.77
**Diastolic BP**		77 [66–88]	76 [65.5–87.5]	80 [67.8–89.5]	0.52
**Pulse**		100 [83.2–113.8]	100 [80–115]	100 [88–110.5]	0.61
**Saturation (RA) (%)**		94 [90–97]	95 [90.5–97]	90.5 [88.8–96]	0.11
**Troponin T** (ng/L)		101 [42.2–252]	94 [34.5–218]	109 [68.2–275.8]	0.27
**NT-proBNP** (pg/mL)		2157 [575–5328]	2424 [585.5–5328]	747.5 [575–4712.2]	0.38
**Creatinine** (mg/dL)		1 [0.8–1.3]	1 [0.8–1.3]	0.9 [0.7–1.4]	0.26
**ALT** (U/L)		21 [15–45.2]	21 [16.8–39.2]	27 [14–61.5]	0.77
**PH**		7.4 [7.3–7.4]	7.4 [7.3–7.4]	7.4 [7.3–7.4]	0.24
**Lactate** (mmol/L)		2.1 [1.3–3.4]	1.8 [1–3.1]	2.3 [1.7–4.9]	0.03
**HB** (g/dL)		13.2 [11.5–14.5]	13.1 [11.5–14.5]	13.4 [11.6–14.6]	0.99
**K (mmol/L)**		4.1 [3.8–4.5]	4.2 [3.9–4.5]	4.1 [3.7–4.4]	0.319
**Mg (mg/dL)**		2.1 [1.9–2.2]	2.1 [1.9–2.3]	2.0 [1.8–9.5]	0.125
**Ca (mg/dL)**		9.1 [8.7–9.5]	9.2 [8.8–9.6]	8.9 [8.6–9.4]	0.102

n(%) unless otherwise indicated; SCAI: Society for Cardiovascular Angiography and Interventions; sPESI: Simplified Pulmonary Embolism Severity Index; BP: blood pressure. RA: room air; NT-proBNP: N-terminal pro-B-type natriuretic peptide; ALT: alanine aminotransferase; PH: potential of hydrogen (acidity/alkalinity); HB: hemoglobin.

**Table 3:** Imaging at baseline.

	Class	Total	Normal QTc	Prolonged QTc	*p*-Value
**N (%)**		95	68 (71.6)	27 (28.4)	
**Computed Tomography**					
** Main PA dilation**		29 (30.9)	19 (28.4)	10 (37)	0.46
** Main PA width**		32 [30–35]	32.5 [30–35]	32 [29.6–33.5]	0.38
** RV/LV ratio change**		56 (60.2)	39 (59.1)	17 (63)	0.82
** Contrast reflux**		49 (55.1)	35 (55.6)	14 (53.8)	1
** Saddle thrombus**		10 (10.5)	6 (8.8)	4 (14.8)	0.46
** Main PA thrombus**		73 (76.8)	52 (76.5)	21 (77.8)	1
** Lobar thrombus**		9 (9.5)	8 (11.8)	1 (3.7)	0.44
** Distal thrombus**		3 (3.2)	2 (2.9)	1 (3.7)	1
**Echocardiography**					
** EF %**		60 [60–60]	60 [60–60]	60 [60–60]	0.27
** RV dilation**	Mild	37 (39.8)	25 (37.3)	12 (46.2)	0.57
	Moderate	29 (31.2)	21 (31.3)	8 (30.8)	
	Severe	2 (2.2)	1 (1.5)	1 (3.8)	
**TAPSE** (mm)		1.6 [1.4–2]	1.6 [1.4–2]	1.6 [1.4–1.8]	0.81
**TAPSV** (cm/s)		9.1 [7–11.3]	9.1 [7–11.1]	10 [5.5–12.2]	0.76
** IVC dilation**		42 (57.5)	28 (58.3)	14 (56)	1
**IVC width** (mm)		14.2 [10–17.4]	14.6 [10.9–16.8]	12.8 [10–18.2]	0.54
** IVC collapse > 50%**		35 (57.4)	17 (47.2)	18 (72)	0.07
** TR**	Mild	44 (49.4)	31 (48.4)	13 (52.0)	0.65
	Moderate	31 (34.8)	24 (37.5)	7 (28.0)	
	Severe	4 (4.5)	2 (3.1)	2 (8.0)	
**TR max** (mmHg)		32 [24–38]	31 [24.1–39]	33 [25.9–37.3]	0.58
**PAP** (mmHg)		45 [40–55]	45 [35–55]	45 [40–60]	0.3
** Septal shift**		56 (60.9)	39 (59.1)	17 (65.4)	0.64

N (%) unless otherwise indicated; PA: pulmonary artery; RV: right ventricle; LV: left ventricle; RV/LV ratio: right ventricular to left ventricular ratio; EF: ejection fraction; TAPSE: tricuspid annular plane systolic excursion; TAPSV: tricuspid annular peak systolic velocity; IVC: inferior vena cava; TR: tricuspid regurgitation; TR max: maximum tricuspid regurgitation pressure; PAP: pulmonary artery pressure.

**Table 4:** Corrected QT measurements.

QTc msec	Total	Normal QTc	Prolonged QTc	*p*-Value
**N (%)**	95	68 (71.6)	27 (28.4)	
** Admission**	463 [441.7–491.5]	450 [431.9–467.9]	500 [491.1–520.2]	<0.001
** Post-treatment**	461.9 [438.9–490.4]	458.8 [436.4–485.1]	471.6 [452.2–510.9]	0.05
** Before discharge**	452.5 [436.4–472.9]	452 [436–470.1]	455.6 [434.8–480.7]	0.439

Median [IQR] unless otherwise indicated. QTc: corrected QT interval; msec: milliseconds.

**Table 5:** Treatment modalities used in patients with PE.

	Total	Normal QTc	Prolonged QTc	*p*-Value
**N (%)**	95	68 (71.6)	27 (28.4)	
**TPA**	9 (9.5)	6 (8.8)	3 (11.1)	0.71
**Catheter direct thrombolysis**	27 (28.4)	23 (33.8)	4 (14.8)	0.08
**Percutaneous aspiration** **thrombectomy**	16 (16.8)	8 (11.8)	8 (29.6)	0.06
**Surgical thrombectomy**	4 (4.3)	3 (4.4)	1 (3.8)	1
**Noninvasive**	46 (48.4)	34 (73.9)	12 (26.1)	0.66

N (%) unless otherwise indicated; TPA—intravenous tissue plasminogen activator.

**Table 6:** In-hospital and post-discharge outcomes by QTc status.

	Total	Normal QTc	Prolonged QTc	*p*-Value
**N (%)**	95	68 (71.6)	27 (28.4)	
**Hospitalization (days)**	6 [4–10.5]	6 [4–10.2]	8 [4.5–11.5]	0.48
**Mortality**				
**In Hospital Mortality**	5 (5.3)	4 (5.9)	1 (3.7)	1
**Mortality Within 3 Months**	5 (5.3)	4 (5.9)	1 (3.7)	1
**Mortality Within 6 Months**	7 (7.4)	4 (5.9)	3 (11.1)	0.4
**Mortality Within 12 Months**	11 (11.6)	8 (11.8)	3 (11.1)	1

N (%) unless otherwise indicated. In-hospital—during index hospitalization; 3 M/6 M/12 M—within 3, 6, 12 months post-discharge; QTc—corrected QT interval.

## Data Availability

The authors affirm that all relevant data supporting the findings of this study are available from the corresponding author upon reasonable request.

## Abbreviations

The following abbreviations are used in this manuscript:

CTA	CT angiography
ECGs	Electrocardiograms
ESC	European Society of Cardiology
msec	millisecond
NT-proBNP	N-terminal pro-B-type natriuretic peptide
PE	Pulmonary embolism
PERT	Pulmonary Embolism Response Team
PVR	Pulmonary vascular resistance
RV	Right ventricular
SCAI	Society of Cardiovascular Angiography and Intervention score
sPESI	Pulmonary Embolism Severity Index
TTE	Transthoracic echocardiography
